# Quercetin-Loaded Ginkgo Starch Nanoparticles: A Promising Strategy to Improve Bioactive Delivery and Cellular Homeostasis in Functional Foods

**DOI:** 10.3390/foods14111890

**Published:** 2025-05-26

**Authors:** Yanyu Sun, Kaiping Cong, Tao Wang, Xiaojing Li, Tingting Li, Gongjian Fan, Dandan Zhou, Caie Wu

**Affiliations:** 1 National Key Laboratory for the Development and Utilization of Forest Food Resources, College of Light Industry and Food Engineering, Nanjing Forestry University, Nanjing 210037, China; 2 Affiliated Cixi Hospital, Wenzhou Medical University, Cixi 315300, China; 3 Department of Chemical Engineering, Xuzhou College of Industrial Technology, Xuzhou 221140, China

**Keywords:** ginkgo nano starch, quercetin, toxicity, apoptosis, antitumor activity

## Abstract

Quercetin (Qc) is a natural bioactive compound derived from plants, with strong anti-inflammatory and antioxidant properties. However, its extreme water insolubility limits its bioavailability and practical utility. To address this, quercetin was encapsulated in ginkgo-derived starch nanoparticles (SNPs) to enhance solubility and stability. In this study, the bioactivity and cellular effects of the SNPs/Qc system were evaluated. Results showed excellent biocompatibility with no toxicity or adverse effects observed in experimental mice. At 25 µg/mL, SNPs/Qc significantly promoted early apoptosis in 3LL cells (33%) and blocked the cell cycle at G1 and G2 phases. The system demonstrated a dose-dependent inhibitory effect on abnormal cell proliferation, with significant activity observed 6 h (hour) post-treatment. Compared with free quercetin, the SNPs/Qc system has dual advantages in improving the bioavailability of quercetin and tumor targeted penetration. After 15 days of ingestion, the survival rate of mice in the SNPs/Qc group increased by 20%, and the tumor volume was only 239 mm^3^, corresponding to a 49.4% decrease. At the same time, specific damage to the cell structure of tumor cells and higher intensity fluorescence accumulation were observed. This study reveals the potential of the SNPs/Qc system as a biocompatible and efficient delivery platform for natural bioactive compounds, particularly in health promotion and functional food applications.

## 1. Introduction

Quercetin (Qc), a natural bioflavonoid with protective properties, is abundantly present in vegetables and fruits, making it a common bioactive component in daily diets [1]. Despite its limited aqueous solubility and poor oral bioavailability, the functional characteristics of quercetin-particularly its antioxidant and anti-inflammatory properties and associated health benefits-have garnered significant attention in recent years [2,3]. Enhancing the solubility and stability of quercetin is critical for its broader development and utilization in the food industry and functional foods. Nano-delivery systems are increasingly prominent in food science, leveraging their tunable particle size and enhanced dispersion properties to improve the delivery efficiency of bioactive compounds [4,5]. Jiang et al. [6] prepared Quinoa starch nanoparticles (QSNPs) to load Qc, and the results indicated that QSNPs enhance quercetin solubility via small particle size (166.25 nm) and high loading (26.62%), improving stability through hydrogen bonding and encapsulation, and retaining 51.58% quercetin after 21 days. Another researcher, Su et al. [5] found that starch nanoparticles can also enhance Qc’s solubility and stability via non-covalent interactions (hydrogen bonding) and encapsulation within a spherical β-glucan network (300 nm diameter), reducing degradation and improving bio-availability. These previous studies strengthen the indications that nanoscale encapsulation strategies for quercetin can significantly enhance its solubility, stability, and bioavailability, substantially expanding its application potential in food processing and functional food development [7,8].

Ginkgo (*Ginkgo biloba* L.), an ancient tree species in China, is renowned for its diverse bioactive compounds and offers nutritional and health-promoting properties [9]. Ginkgo seeds contain approximately 68% starch and have been utilized as both food and traditional medicine for thousands of years [10]. Ginkgo extracts have been employed in the treatment of diabetes, cancer, and thrombosis [11]. Wang et al. [12] proved that ginkgo seeds are better than corn in the preparation of starch nano-spheres (SNPs). The capacity of Qc loaded with SNPs was higher than that of corn starch. Qc could be continuously released in simulated gastric juice and significantly inhibit the growth of A549 cells [13].

To explore the in vivo safety of the SNPs/Qc delivery system for functional food applications, Institute of Cancer Research (ICR) mice were used as test subjects for comprehensive evaluation through oral acute and subacute toxicity experiments [14]. The assessment focused on key health indicators relevant to food safety, including changes in body weight (BW), biochemical parameters, hematological examinations, organ coefficients, and histopathological indicators [15]. Building upon the aforementioned toxicological experiments, we have, for the first time, investigated the effects of quercetin (Qc) on cancer cells. Reportedly, Qc could induce cell apoptosis via the mitochondrial pathway [16]. Guo et al. [17] have demonstrated that, after 24 h of Qc treatment, the viability of A549 and H1299 cells was inhibited, and mitochondria-dependent apoptosis was induced. To compare the tumor cell inhibitory activity of SNPs/Qc in vitro, F127 (EO100-PO65-EO100)/Qc was utilized [18,19].

In our previous research [13], we successfully prepared SNP/Qc complexes with a maximum loading capacity of 1140 μg Qc/mg SNP (24 mM Qc, 250 min). Dynamic light scattering (DLS) revealed that unloaded SNPs had a hydrodynamic diameter of 80 ± 8.23 nm (polydispersity index, PDI = 0.34), increasing to 165 ± 9.65 nm (PDI = 0.28) post-loading. In vitro release studies revealed that SNP/Qc exhibited pH-dependent sustained release kinetics. Free Qc exhibited rapid release (>80% within 1 h in pH 2.0 gastric fluid), whereas SNP/Qc displayed a triphasic release profile: burst release (28.1% at pH 2.0/0.6 h; 32.5% at pH 6.8/2 h), sustained release (59.5–66.7%), and slow degradation-driven release (71.3–78.2% at 12 h). Notably, SNP/Qc demonstrated dose-dependent cytotoxicity, achieving 55.16% inhibition of A549 cells at 200 μg/mL, significantly surpassing free Qc. Therefore, in this study, we continue to investigate the mechanism by which the SNPs/Qc system inhibits tumor cell activity by assessing membrane potential and cell cycle progression. Furthermore, recognizing that animal metabolism, circulatory dynamics, and fluid regulation during natural growth and activity may influence the efficacy of bioactive compounds, we further evaluated the anti-tumor effects of orally administered SNPs/Qc in nude mice [20]. This research not only elucidates the potential mechanisms underlying the SNPs/Qc system but also provides a scientific foundation for its application in functional foods.

## 2. Materials and Methods

### 2.1. Chemicals and Reagents

SNPs/Qc was supplied by the National Key Laboratory for the Development and Utilization of Forest Food Resources, Nanjing Forestry University, Nanjing, Jiangsu, China [13].

Heparin sodium and paraformaldehyde (Nanjing zebra Co., Nanjing, China). Various chemicals and reagents, including quercetin and ginkgo seeds (KeyGEN Biochemical Co. Nanjing, China). DMSO (SIGMA Co., Ltd., Burbank, CA, USA). DMEM medium (12800-082) (GIB-CO Co., Ltd., Grand Island, NY, USA). Fetal bovine serum (FBS500) (ExCell Biology Co., Ltd., Mountain View, CA, USA). Lung cancer 3LL cell line (Chinese Academy of Sciences, Shanghai, China). All other reagents used were of analytical grade.

### 2.2. Animals

Male special pathogen free (SPF) grade BALB/c-nu mice weighing 18 to 22 g and aged 4 to 5 weeks, were purchased from Nanjing Junke Biological Co., Ltd. (Nanjing, China). After one week of acclimation, mice were randomly divided into three or four groups with five animals per group. Standard rodent maintenance food and drinking water were provided ad libitum. All mice were fed in cages (5 animals each) and housed in an environment with controlled temperature (22.0 ± 1.0 °C) and humidity (50 ± 10%) and a 12-h light–dark cycle. All experimental procedures were conducted according to the institutional guidelines for the care and use of laboratory animals of the Nanjing Ogpharmacetical Co., Ltd. (Nanjing, China) [Approval Number: SYXK (Su) 2017-0017]. The protocol for these experiments was approved by the Experimental Animal Welfare and Ethics Committee of Nanjing Forestry University (NJFU-2017031209).

### 2.3. Acute Oral Toxicity Test

Mice were randomly divided into four groups, including one control group and three test groups. SNPs/Qc test doses were 20, 100, and 500 mg/kg BW, respectively. Control mice received sterile saline alone. Three groups were administered SNPs/Qc by oral gavage.

### 2.4. Subacute (30 d) Toxicity Test

Twenty mice were randomly assigned to one control group and three test groups. SNPs/Qc test doses were 20, 100 and 500 mg/kg BW, respectively. After 30 d (day) of feeding, the physiological characteristics of mice were observed and recorded daily. BW of mice was recorded at the same time every week. At the end of the experiment, blood was drawn from the mice’ orbits and collected into a 2 mL centrifuge tube (with anticoagulant) to determine hematology (lymphocyte count (LYC), red blood cell count (RBC), neutrophils count (NE), hemoglobin concentration (HGB), hematocrit (HCT), mean corpuscular volume (MCV), mean corpuscular hemoglobin (MCH) and white blood cell count (WBC)) and biochemical analyses (alanine transaminase (ALT), aspartate transaminase (AST), total serum protein (TP), albumin (ALB), albumin/globulin ratio (A/G), blood glucose (GLU), total bilirubin (TBIL), total cholesterol (CHOL) and triglycerides (TG)) [21,22].

Following a 30-d feeding trial, mice were humanely euthanized via cervical dislocation under isoflurane anesthesia for systematic necropsy and pathological changes in visceral organs were examined. The vital organs from each mouse were removed and weighed. Ratios of each organ to final BW were calculated [23,24]. Weighed organs were cut into small pieces and completely immersed in 4% paraformaldehyde for fixation. After fixation, collected samples were prepared and stained with hematoxylin and eosin (H&E) to observe histopathological changes [25].

### 2.5. Detection of Apoptosis by Annexin V-APC/7-AAD Double-Staining

According to the results of cell inhibition experiments (Table S1) [13], 3LL cells were selected as follow-up experimental cells with a higher inhibition rate. In the logarithmic growth phase, 3LL cells were digested and inoculated into six-well plates overnight. After 48 h of SNPs/Qc or F127/Qc treatment, cells were trypsinized and washed with phosphate buffer (PBS). The doses of SNPs/Qc and F127/Qc were 6.25 μg/mL, 12.50 μg/mL and 25.00 μg/mL, respectively. Amounts of 5 μL Annexin V-APC and 5 μL 7-AAD were added to the cell suspension. Stained cells were incubated for about 10 min at room temperature in the dark and analyzed by flow cytometry [26,27].

### 2.6. Cell Cycle Analysis

Cells were collected by gentle trypsinization, seeded in six-well plates, and incubated for 1 d. Subsequently, cells were trypsinized with three doses of SNPs/Qc and F127/Qc. On completion of the culture, cells were washed with PBS and added to 100 μL RNase A in a 37 °C water bath for 30 min. After treatment, cells were added to 400 μL propidium iodide (PI) at 4 °C for 30 min and detected red fluorescence intensity at 488 nm.

### 2.7. Measurement of Mitochondrial Membrane Potential

After being washed with PBS and centrifuged at 2000 rpm for 5 min, cells were adjusted to a concentration of 1 × 10^6^ cells/mL. JC-1 working solution was prepared by adding 1 μL JC-1 into the 500 μL 1× incubation buffer. Cells were added to 500 μL JC-1 working solution and incubated at 37 °C with 5% CO_2_ for 15–20 min. Then, cells were washed twice with 1× incubation buffer, resuspended in 500 μL 1× incubation buffer and immediately analyzed for their mitochondrial membrane potential [28,29].

### 2.8. Mice Lung-Cancer-Inoculated Tumor Models and Animal Groups

The 3LL cells were cultured in a DMEM medium at 37 °C and 5% CO_2_. The cell growth status was observed and the culture medium was changed regularly. After continuous culture, 3LL cells were collected and counted. Then, 3LL cell concentration was adjusted to 1 × 10^7^ cells/mL by adding culture solution. The 0.1 mL 3LL lung cells were injected subcutaneously into the back of anesthetized mice. The tumor growth of mice was closely monitored. After 5 d of tumor cell inoculation, tumor-bearing mice were randomized into three groups with 5 mice in each group. The mice were gavaged with 10 μg/g Qc or 20 μg/g SNPs/Qc daily for 15 d. The mice were euthanized on d 16, and tumor tissues were collected for analysis.

### 2.9. Volume Measurement and H&E-Stained Sections of Tumor Tissues

Tumor volumes were measured every day using a caliper and calculated by a standard formula (length × width^2^ × 0.5) [30]. The largest sections of tumor tissues were fixed in 10% formalin and stained by H&E to observe tumor cells [21,31].

### 2.10. Fluorescence Imaging Analysis of the Living Body and Organs of Tumor-Bearing Mice

After staining SNPs/Qc with fluorescent dyes, tumor-bearing mice were gavaged at normal dosage. At 10 min, 20 min, 30 min, 1 h, 2 h, 4 h, 6 h and 8 h, treated mice were observed and captured by in vivo imaging camera of ultra-high resolution (CRI Maestro 2) [32] and tumor-bearing mice were sacrificed after 6 h for dissection to capture tumor tissues and livers.

### 2.11. Statistical Analysis

Five parallel tests were conducted for all experiments, and values are expressed as the mean ± SD. One-way ANOVA was conducted, and the significance level was defined as *p* < 0.05. Statistical analyses were performed using SPSS 26.0 software (IBM, Armonk, NY, USA).

## 3. Results

### 3.1. Acute and Subacute Toxicity

#### 3.1.1. BW and Organ Coefficient

**Figure 1:**
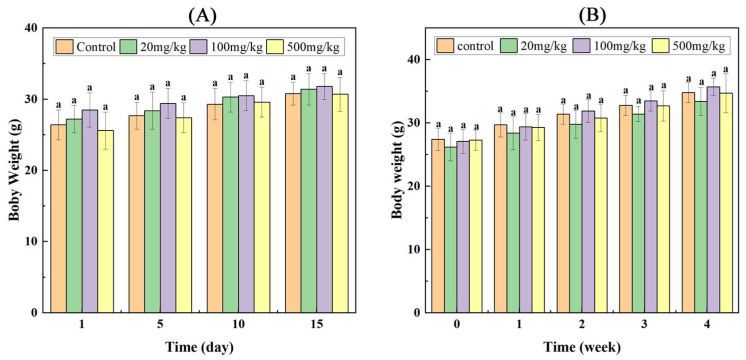
Mean body weights of mice treated with different doses of SNPs/Qc. BW of mice treated by oral toxicity test of SNPs/Qc system for 15 d (**A**) and 4 w (**B**). Each value represents the mean of three replicates, and error bars indicate standard deviations (±SD). Lowercase letter (a), in the graph, indicates no significant differences between control and treatment groups at the same administration time (*p* < 0.05).

In acute and subacute toxicity tests, mice grew normally without mortality. No significant difference was observed in skin color, BW (Figure 1), behavioral activities and defecation of mice between experimental and control groups. Additionally, an increased tendency of BW was observed during the experimental period.

In addition, compared with the control group, the data associated with organ coefficients (Table 1) showed that there was no significant difference (*p* > 0.05) in the ratios of heart/BW, liver/BW, lung/BW, kidney/BW and spleen/BW of the mice in all of the experimental groups.

#### 3.1.2. Histopathological Examination

**Figure 2:**
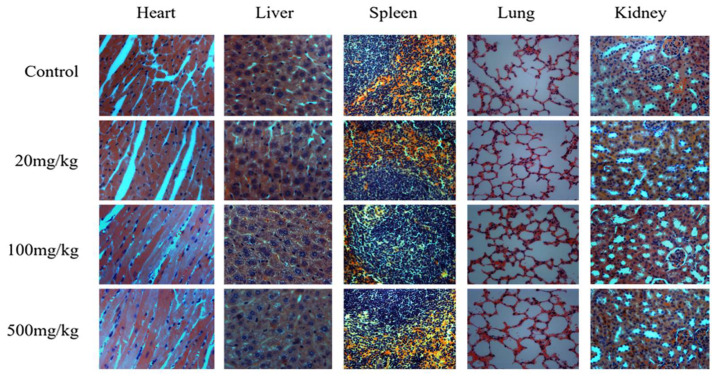
Histopathological examination of the mice’ heart, kidney, lung, liver and spleen tissue treated with SNPs/Qc in the subacute (20, 100, and 500 mg/kg) toxicity test. H&E coloring.

According to the observation of tissue sections (Figure 2), compared with the control, general appearances and internal organs of treated mice showed normal structure, size, and color. There was no edema, necrosis or inflammation in the organs. The histopathological examination revealed closely arranged myocardial fibers and cells in the hearts, normal cell nucleus in the liver, clearly visible red and white pulp in the spleen, uniform and regular alveolar tissue in the lung, and clear structure in the kidney.

#### 3.1.3. Hematological and Biochemical Parameters

**Figure 3:**
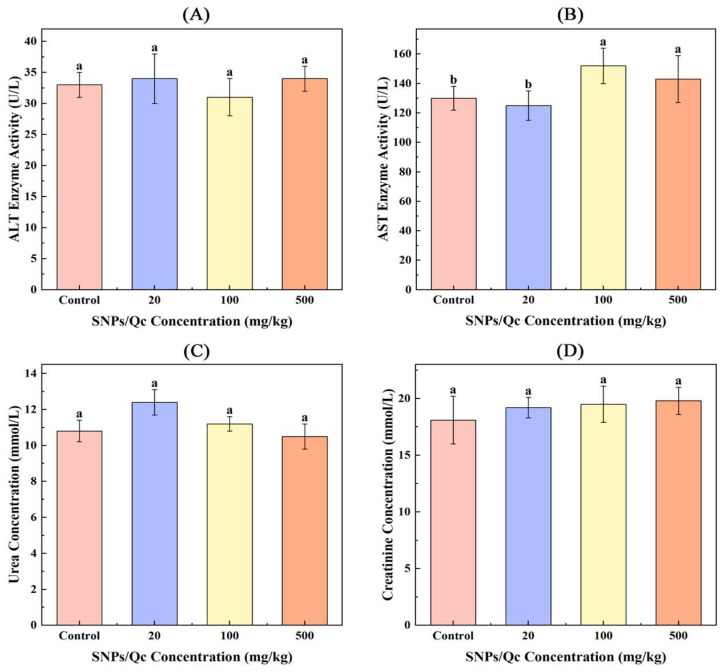
Effects of SNPs/Qc on liver and kidney function in mice. (**A**) The enzyme activity of ALT (**A**) and AST (**B**) in the plasma of mice in the control and administration groups. The content of creatinine (**C**) and urea (**D**) in mice in the control and administration groups. Each value represents the mean of three replicates, and error bars indicate standard deviations (±SD). Different lowercase letters (a, b), in the graph, indicate significant differences among four experimental groups at the same administration time (*p* < 0.05).

As can be seen from Figure 3, there was no significant difference in ALT, AST, urea and creatinine between the experimental and the control groups (*p* > 0.05). Table 2 represents the hematological and biochemical parameters of mice treated with control and SNPs/Qc in the acute and subacute toxicity tests. There was a little increase in lymphocytes, HMB, MCV and WBC and a decrease in NE compared with the control. The results of this study show a decrease in CHOL and ALB (20 mg/kg), TBIL and TG (20, 100 and 500 mg/kg) and an increase in GLO and TP (500 mg/kg).

### 3.2. Cancer Cell Inhibitory Activity of SNPs/Qc

#### 3.2.1. Effects of SNPs/Qc or F127/Qc on Apoptosis of 3LL Cells

**Figure 4:**
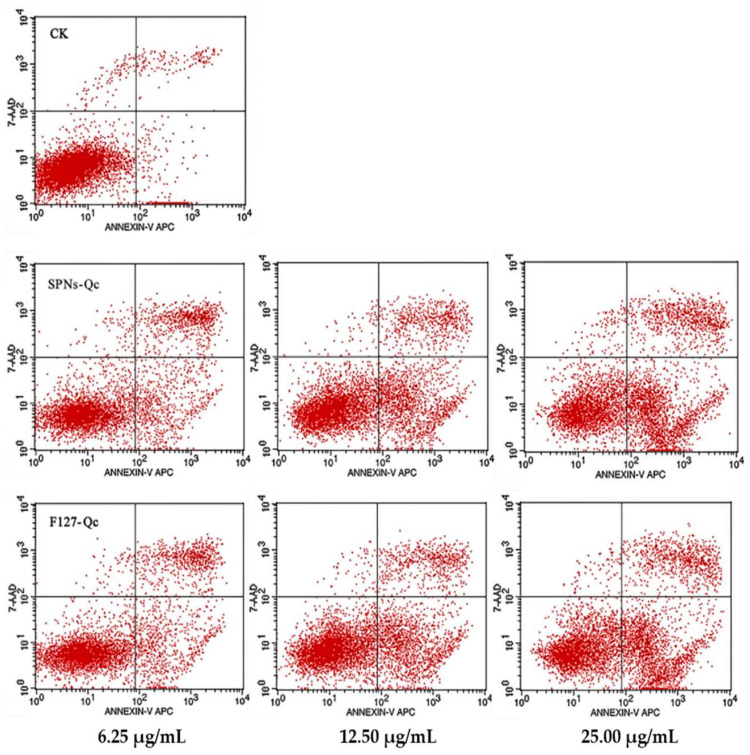
Effects of SNPs/Qc and F127/Qc on apoptosis of 3LL cells. The lower left, lower right, upper right and upper left quadrants respectively represent living cells, early apoptotic cells, late apoptotic cells and necrotic cells.

As can be seen in Figure 4, the apoptosis rate of SNPs/Qc groups was significantly higher than that of the control group after administration (*p* > 0.05). After treatment with 6.25, 12.5, and 25 μg/mL of SNPs/Qc, the apoptosis rates were respectively 21.47 ± 2.93%, 28.78 ± 2.37%, and 37.05 ± 1.91%, in a dose-dependent manner (Table S2).

#### 3.2.2. Effects of SNPs/Qc or F127/Qc on the Cell Cycle of 3LL Cells

**Figure 5:**
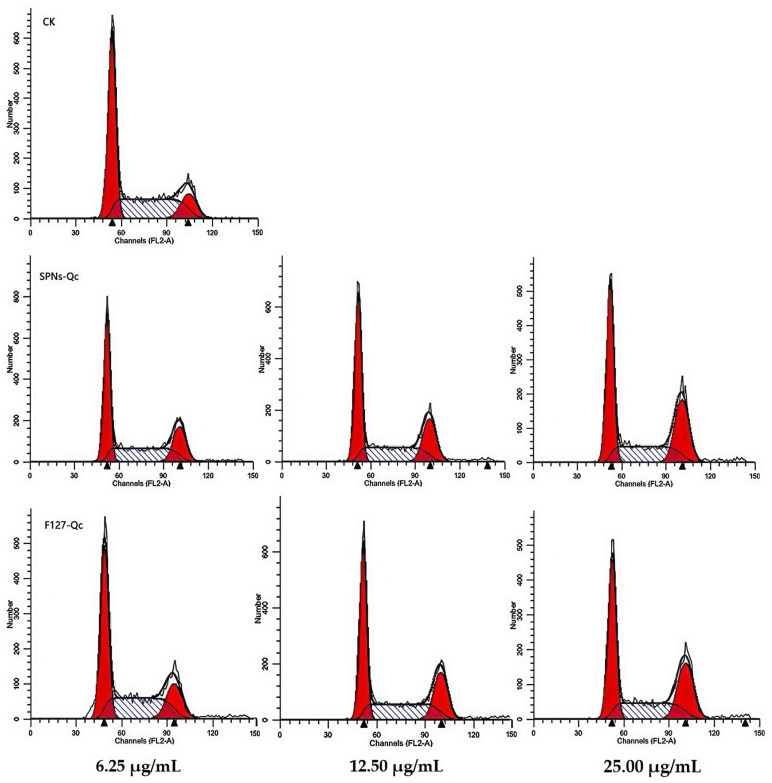
Effects of SNPs/Qc and F127/Qc on the cell cycle of 3LL cells. The red peaks on both sides indicate that cells were in G1 and G2 phases. The middle peak indicates that cells were in the S phase.

Compared with the control, the cell cycle of 3LL cells was significantly affected by SNPs/Qc and F127/Qc nanocomposites (Figure 5). The proportion of 3LL cells in the G2 phase gradually increased with the increase of SNPs/Qc dose. The proportion of G2 cells was respectively increased to 23.84 ± 0.54% at the dose of 25 μg/mL (Table S3).

#### 3.2.3. Effects of SNPs/Qc or F127/Qc on the Mitochondrial Membrane Potential of 3LL Cells

**Figure 6:**
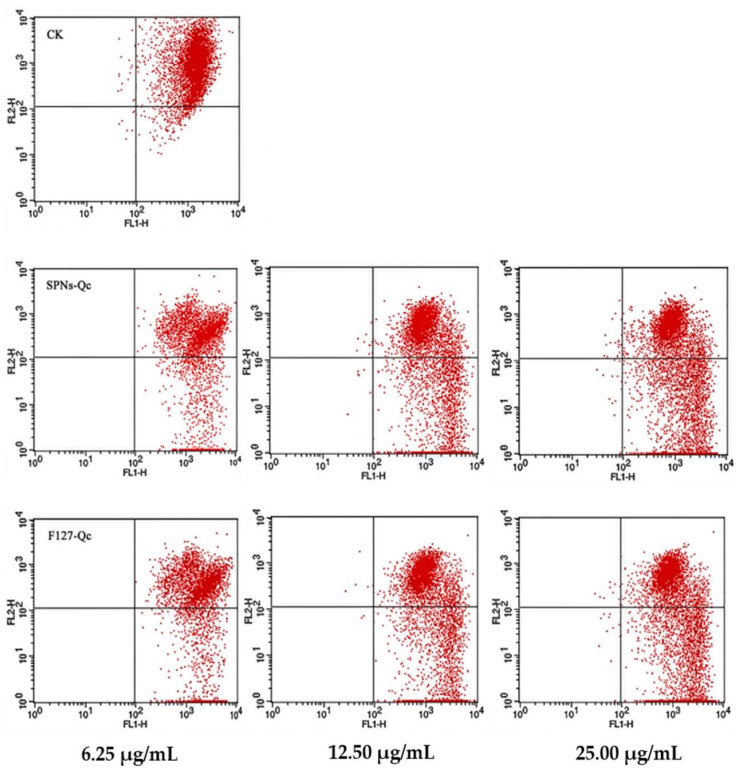
Effects of SNPs/Qc and F127/Qc on the mitochondrial membrane potential of 3LL cells. The lower right quadrant in the figure represents the proportion of positive cells with reduced mitochondrial membrane potential.

The changes in mitochondrial membrane potential in 3LL cells after SNPs/Qc or F127/Qc treatment are shown in Figure 6. Compared with the control, the SNPs/Qc and F127/Qc nanocomposites can significantly change the mitochondrial membrane potential of 3LL cells. The results show that the percentages of positive cells were as high as 42.4 ± 1.92% in the treatment groups, while the mean cells were 3.72 ± 0.21% in the control (Table S4).

### 3.3. Biological Activity of SNPs/Qc

#### 3.3.1. BW, Survival Rate and Tumor Volume of Tumor-Bearing Mice

**Figure 7:**
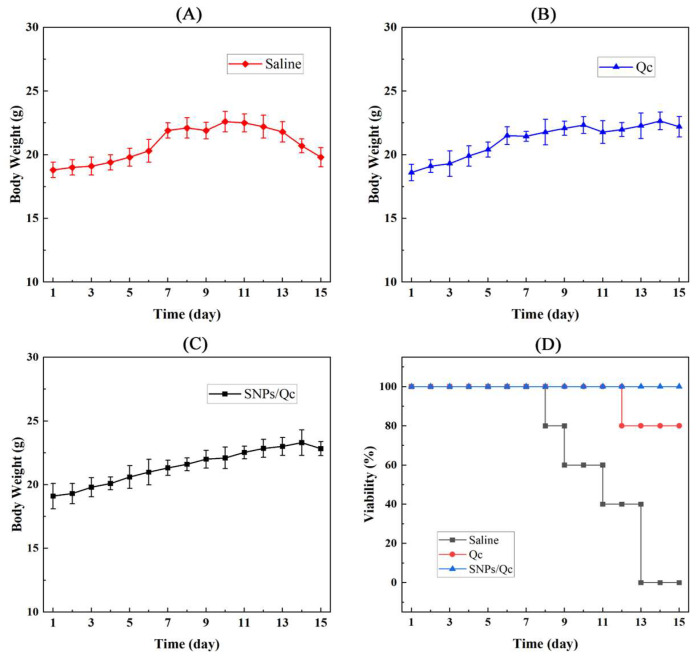
Effects of Qc and SNPs/Qc on the body weight and survival rate of tumor-bearing mice. BW of tumor-bearing mice in the control group (**A**), the administration group with 10 μg/g Qc (**B**) and 20 μg/g SNPs/Qc (**C**). (**D**) The survival rate of tumor-bearing mice in the control and administration groups. Each value represents the mean of three replicates, and error bars indicate standard deviations (±SD).

None of the mice in the three groups exhibited abnormal BW (Figure 7). However, the mice in the control lost weight on day 13. The survival time was recorded as 8–13 d in the control. In the Qc group, the survival rate after the experiment was 80%. After 15 d of nanoparticle ingestion, there was no death in SNPs/Qc group (Figure 7D).

**Figure 8:**
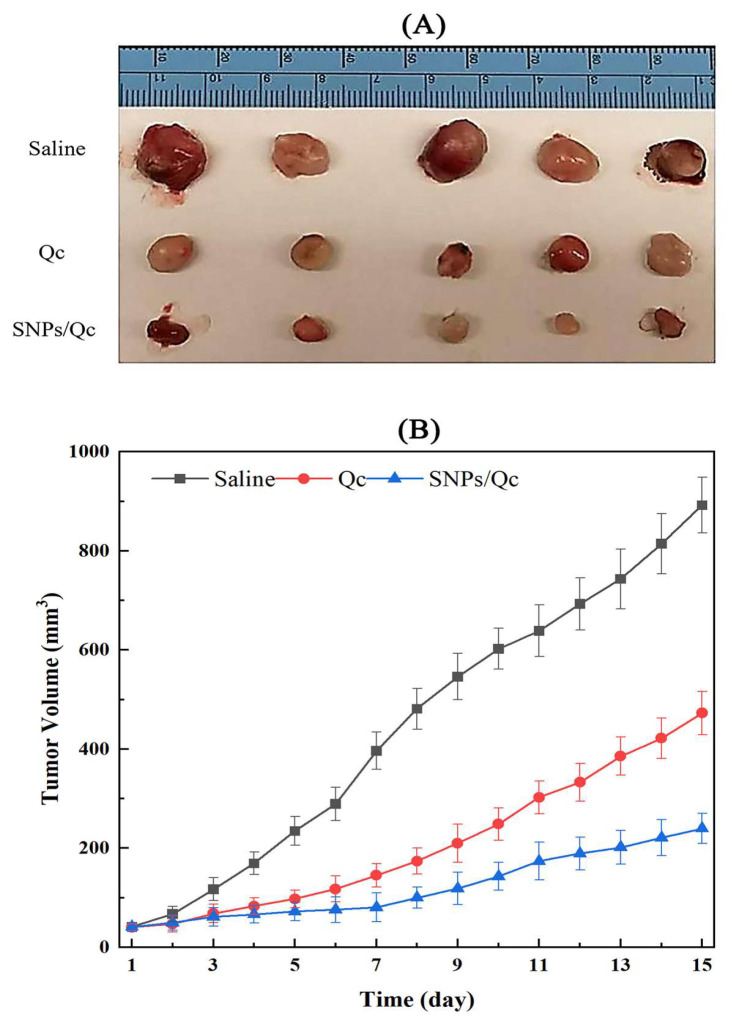
Effects of Qc and SNPs/Qc on the tumor volume change of tumor-bearing mice. (**A**) Tumor pictures were obtained from the anatomy of tumor-bearing mice at the end of the experiment. (**B**) Tumor volume change in the control and administration groups. Each value represents the mean of three replicates, and error bars indicate standard deviations (±SD).

As is shown in Figure 8, the tumor volumes of mice in Qc and SNPs/Qc groups were significantly reduced (*p* < 0.01) when compared with the control. After 15 d of nanoparticle ingestion, tumor volumes of mice in the control grew to about 892 mm^3^, while tumor volumes in Qc and SNPs/Qc groups were 472 mm^3^ and 239 mm^3^, respectively.

#### 3.3.2. Observation of Tumor Tissue Sections

**Figure 9:**
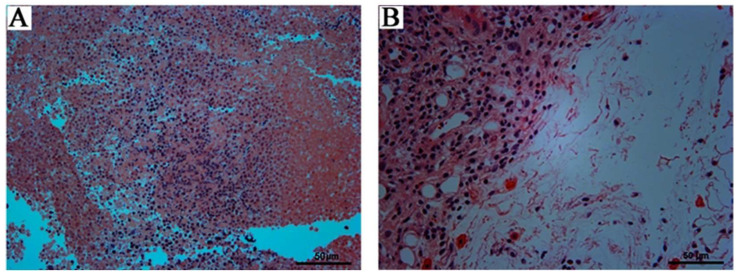
Effects of SNPs/Qc on the tumor tissue of tumor-bearing mice. The staining section of tumor tissue in the control group (**A**) and the administration group with 20 μg/g SNPs/Qc (**B**).

The stained sections of tumor tissue in the control and SNPs/Qc groups are shown in Figure 9. Specifically, when compared with the SNPs/Qc group, the cells of tumor tissue without treatment were relatively neat and close. Furthermore, tumor tissue cells in the SNPs/Qc-treated group exhibited numerous structurally disrupted intercellular gaps, suggesting enhanced permeability and potential nanoparticle accumulation.

#### 3.3.3. Pharmacokinetic In Vivo Tumor and Fluorescence Imaging of Tumor-Bearing Mice

**Figure 10:**
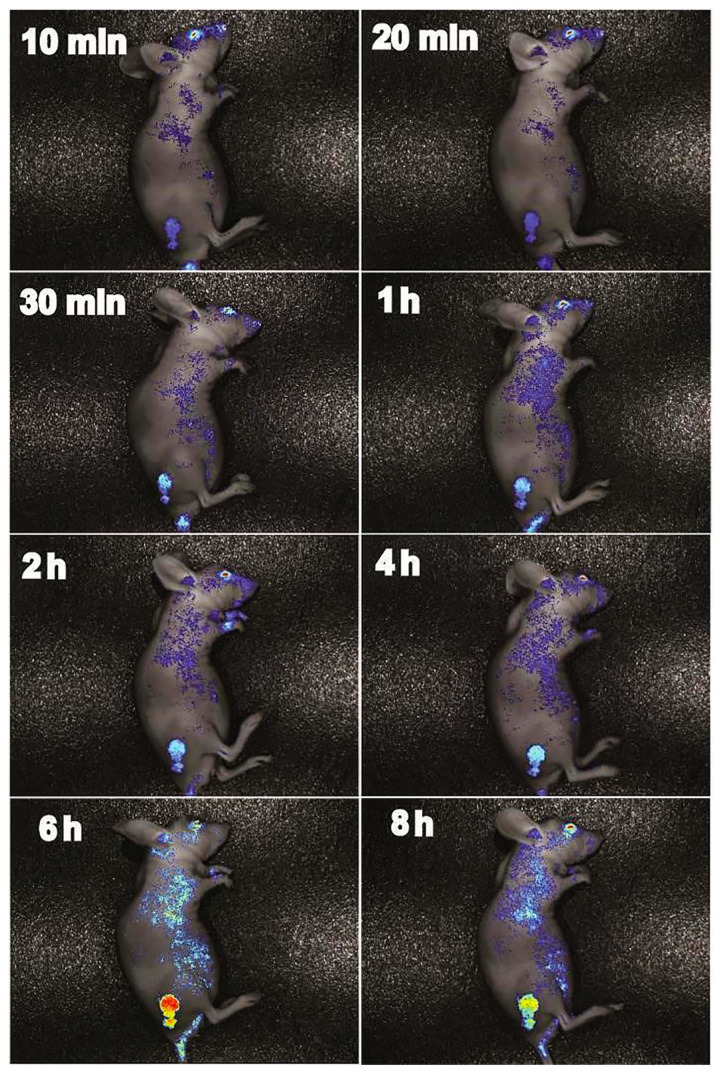
In vivo fluorescence images of pharmacokinetics in tumor-bearing mice.

**Figure 11:**
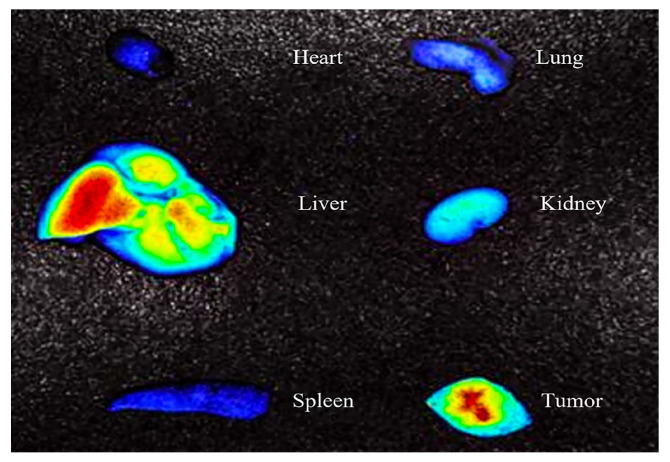
In vivo fluorescence images of organs and the tumor in tumor-bearing mice. Observe the organs, including the heart, kidney, lung, liver and spleen.

Figure 10 showed the distribution of SNPs/Qc in mice. After 6 h of administration, the mice were dissected to observe the nanoparticles aggregation in each organ in Figure 11. Oral SNPs/Qc successfully reached and accumulated in the tumor site. After 1 h of administration, no obvious nanoparticles were detected at the tumor site. The strongest fluorescence intensity of the tumor site was captured at 6 h, and fluorescence mainly accumulated in the liver and tumor of mice.

## 4. Discussion

As a food-grade carrier, SNPs represent a metabolizable carbohydrate that is ultimately catabolized via physiological pathways into energy, CO_2_, and water. Consistent with previous studies on dietary polyphenols, Qc has been established as a safe bioactive compound [33]. Zhao et al. [34]. confirmed its safety through acute exposure and developmental toxicity assessments, consistent with the Generally Recognized as Safe (GRAS) profile of plant-derived phytochemicals. Notably, male mice fed Qc at 3000 mg/kg body weight (BW)/day for 28 d showed no significant alterations in nutritional safety indicators compared with controls. While both components exhibit inherent safety, their combined metabolic behavior within the encapsulation system necessitated evaluation [35]. Acute and subacute exposure trials revealed no observable adverse effects, confirming the complete biodegradability of the SNPs-Qc nanocomplex in vivo. A transient elevation of serum aspartate aminotransferase (AST) levels was observed in medium–high-dose groups during the intervention period, with hepatic accumulation of bioactive components indicating tissue-specific metabolic handling [36]. These fluctuations are indicative of adaptive physiological responses during bioactive compound metabolism, where AST release coincides with transient hepatic metabolic activation as opposed to signifying hepatotoxicity [37]. This interpretation is further corroborated by histopathological analyses revealing no structural abnormalities in major organs across treatment groups. The observed AST dynamics likely correspond to a self-regulatory mechanism maintaining metabolic equilibrium during bioactive compound utilization [38]. Collectively, these findings validate that the SNPs/Qc system functions within the bounds of physiological adaptability, reinforcing its suitability as a food-grade delivery platform.

Quercetin has been shown to modulate cellular signal transduction, potentially influencing apoptosis-related pathways [36]. The regulatory mechanism of the SNPs/Qc system on cellular homeostasis was investigated through mitochondrial membrane potential assessment and cell cycle progression analysis. The results indicate that SNPs/Qc can significantly increase the proportion of early apoptotic cells, arrest the cell cycle, and reduce mitochondrial membrane potential, suggesting its potential in maintaining cellular homeostasis. The balance between anti-apoptotic and pro-apoptotic proteins, such as Bcl-2 and Bax, plays a crucial role in determining cellular responses [39,40]. Previous studies have highlighted that cells with a lower Bcl-2/Bax ratio are more prone to apoptosis [41,42]. Experimental data showed that SNP/Qc treatment induced apoptosis in 3LL cancer cells, and subsequent analysis showed that Qc reduced the expression rate of Bcl-2/Bax. When cells receive apoptotic signals, Bax proteins translocate from the cytoplasm to the mitochondrial membrane, forming channels that decrease mitochondrial membrane potential and increase permeability [43]. This mechanism underscores the potential of the SNPs/Qc system as a functional food ingredient for promoting cellular health.

After inoculating 3LL cells, the diet, metabolism, and other physical functions of mice may be affected by the increase in tumor volume, thereby reducing the weight of the mice. Compared with the control, the tumor volumes of mice were significantly reduced by SNPs/Qc intervention for 15 d. Qc successfully reached the tumor site through the metabolic cycle and played a significant role in inhibiting the growth of tumor cells [44]. The experimental results show that the anti-tumor effect of SNPs/Qc is significantly better than the free Qc. Three factors could account for this result. Firstly, Qc exhibits enhanced performance when encapsulated within starch nanoparticles, owing to the improved dispersion, stability, and bioavailability conferred by the nano load delivery system. Secondly, the efficacy of Qc may be compromised by its limited aqueous solubility and chemical instability under the environment of multiple enzymes and electrolytes in the organism [45]. Thirdly, because of the interaction between the charges of Qc and the cell surface, Qc could not successfully enter the cells. SNPs/Qc could reduce the barriers stopping Qc from entering cells [46].

After 6 h of ingestion, the accumulation of fluorescent compounds in the liver of mice indicated that certain bioactive food ingredients or their delivery markers were absorbed through digestive and metabolic pathways [47]. Notably, there was no significant aggregation of fluorescence in other organs. Although in vivo bioavailability studies and organ-specific fluorescence imaging at 8 h post-ingestion do not fully account for the localized presence of Qc or SNPs/Qc in the liver and tumor regions, the observed substantial reduction in tumor volume and improved survival rates suggest that the fluorescence aggregation is primarily due to the efficient accumulation of SNPs/Qc in these tissues [48]. Overall, when incorporated into functional food formulations, SNPs/Qc nanoparticles demonstrate significantly better anti-tumor effects compared with free Qc, underscoring their potential as an effective nutraceutical ingredient for food-based interventions.

## 5. Conclusions

In summary, the effects of SNPs/Qc on cell apoptosis, cell cycle regulation, and tumor inhibition were investigated in this work, with a view toward their potential application in functional foods and nutraceuticals. No obvious toxic or side effects were observed in SNPs/Qc nanoparticles based on acute and subacute toxicity assessments. SNPs/Qc demonstrated significant inhibitory activity on 3LL cells, notably increasing the proportion of cells in the G1 and G2 phases, and substantially altering the mitochondrial membrane potential, as evidenced by the elevated proportion of positive cells. These changes contributed to the partial apoptosis of tumor cells and inhibited tumor growth, ultimately prolonging the lifespan of tumor-bearing mice. These findings suggest that an appropriate concentration of SNPs/Qc could effectively replace water-insoluble free Qc while maintaining its functional benefits. SNPs/Qc nanoparticles, characterized by unique physicochemical properties and excellent biocompatibility, establish a theoretical foundation for incorporating bioactive compounds into health-promoting foods and present innovative strategies for their development and application within the food industry.

## Figures and Tables

**Table 1:** Relative organ weight (g/g of BW) of mice treated with SNPs/Qc nanocomposites in oral subacute toxicity.

Parameters	Heart (%)	Liver (%)	Spleen (%)	Lungs (%)	Kidney (%)
Control	0.48 ± 0.04 ^a^	4.14 ± 0.15 ^a^	0.32 ± 0.08 ^a^	0.83 ± 0.10 ^a^	1.41 ± 0.22 ^a^
20 mg/kg	0.52 ± 0.08 ^a^	3.86 ± 0.18 ^a^	0.34 ± 0.06 ^a^	0.85 ± 0.09 ^a^	1.34 ± 0.14 ^a^
100 mg/kg	0.54 ± 0.11 ^a^	4.24 ± 0.31 ^a^	0.31 ± 0.08 ^a^	0.79 ± 0.06 ^a^	1.37 ± 0.11 ^a^
500 mg/kg	0.51 ± 0.06 ^a^	4.21 ± 0.22 ^a^	0.35 ± 0.05 ^a^	0.76 ± 0.07 ^a^	1.43 ± 0.15 ^a^

Values in the same lowercase letter within a column are not significantly different (*p* > 0.05).

**Table 2:** Hematological and biochemical parameters of mice treated with SNPs/Qc nanocomposites.

Parameters	Control	20 mg/kg	100 mg/kg	500 mg/kg
Hematological parameters of mice				
HCT (LL)	0.51 ± 0.08 ^a^	0.55 ± 0.09 ^ab^	0.58 ± 0.03 ^b^	0.54 ± 0.04 ^ab^
HGB (g/L)	151.00 ± 8.00 ^a^	158.00 ± 9.00 ^ab^	159.00 ± 7.00 ^b^	162.00 ± 9.00 ^b^
LYC (109/L)	2.75 ± 0.18 ^a^	3.46 ± 0.89 ^b^	2.88 ± 0.50 ^a^	2.96 ± 0.41 ^a^
MCH (pg)	14.50 ± 0.60 ^a^	14.90 ± 0.90 ^a^	15.10 ± 0.70 ^a^	14.80 ± 0.40 ^a^
MCV (fL)	52.70 ± 1.10 ^a^	52.50 ± 1.90 ^a^	53.40 ± 1.30 ^a^	53.10 ± 1.40 ^a^
NE (109/L)	0.45 ± 0.08 ^a^	0.43 ± 0.09 ^a^	0.39 ± 0.07 ^a^	0.41 ± 0.14 ^a^
RBC (1012/L)	10.52 ± 0.58 ^a^	10.48 ± 0.69 ^a^	10.75 ± 0.43 ^a^	10.89 ± 0.77 ^a^
WBC (109/L)	2.35 ± 0.22 ^a^	3.75 ± 1.09 ^b^	2.77 ± 0.49 ^a^	2.85 ± 0.38 ^a^
Biochemical parameters of mice				
ALB (g/L)	35.10 ± 4.80 ^a^	34.80 ± 3.90 ^a^	36.90 ± 4.70 ^a^	36.20 ± 2.90 ^a^
CHOL (mmol/L)	5.35 ± 0.12 ^a^	4.95 ± 0.61 ^b^	5.77 ± 0.48 ^a^	5.85 ± 0.43 ^a^
GLOB (g/L)	23.54 ± 1.58 ^a^	22.88 ± 2.69 ^a^	22.74 ± 2.43 ^a^	23.69 ± 3.27 ^a^
TBIL (μmol/L)	4.50 ± 1.20 ^a^	4.30 ± 0.90 ^a^	3.90 ± 0.80 ^a^	4.10 ± 0.60 ^a^
TG (mmol/L)	1.78 ± 0.23 ^a^	1.54 ± 0.43 ^b^	1.64 ± 0.37 ^ab^	1.75 ± 0.17 ^a^
TP (g/L)	59.70 ± 1.80 ^a^	58.30 ± 1.90 ^a^	58.40 ± 1.50 ^a^	60.10 ± 1.40 ^b^

Values within a row marked with different lowercase superscript letters (a, b) differ significantly (*p* < 0.05), while common letters indicate no statistically significant differences (*p* > 0.05).

## Data Availability

The original contributions presented in the study are included in the article/Supplementary Materials; further inquiries can be directed to the corresponding author.

## Supplementary Materials

The following supporting information can be downloaded at https://www.mdpi.com/article/10.3390/foods14111890/s1, Table S1: IC50 of Qc, SNPs/Qc and F127/Qc on five kinds of cancer cells; Table S2: Effect of SNPs/Qc and F127/Qc on 3LL cell apoptosis; Table S3: Effect of SNPs/Qc and F127/Qc on 3LL cell cycle; Table S4: Effect of SNPs/Qc and F127/Qc on mitochondrial membrane potential of 3LL cell.

## Abbreviations

The following abbreviations are used in this manuscript:

A/G	Albumin/globulin ratio
ALB	Albumin
ALT	Alanine transaminase
AST	Aspartate transaminase
BW	Body weight
CHOL	Total cholesterol
GLU	Blood glucose
HCT	Hematocrit
H & E	Hematoxylin and eosin
HGB	Hemoglobin concentration
ICR	Institute of Cancer Research
LL	Lower left
LR	Lower right
LYC	Lymphocyte count
MCH	Mean corpuscular hemoglobin
MCV	Mean corpuscular volume
NE	Neutrophils count
Qc	Quercetin
RBC	Red blood cell count
SNPs	Starch nanoparticles
SNPs/Qc	Starch nanoparticles/quercetin
TBIL	Total bilirubin
TG	Triglycerides
TP	Total serum protein
UL	Upper left
UR	Upper right
WBC	White blood cell count

## References

[1] Giuliani C., Di Dalmazi G., Bucci I., Napolitano G. (2024). Quercetin and Thyroid. Antioxidants.

[2] Moon J.H., Eo S.K., Lee J.H., Park S.Y. (2015). Quercetin-induced autophagy flux enhances TRAIL-mediated tumor cell death. Oncol. Rep..

[3] Lawson M.K. (2023). Improvement of Therapeutic Value of Quercetin with Chitosan Nanoparticle Delivery Systems and Potential Applications. Int. J. Mol. Sci..

[4] Hosseini-Ashtiani N., Tadjarodi A., Zare-Dorabei R. (2021). Low molecular weight chitosan-cyanocobalamin nanoparticles for controlled delivery of ciprofloxacin: Preparation and evaluation. Int. J. Biol. Macromol..

[5] Su Y., Zhou Q., Xu H., Huang M., Li S., He J., Cheng K.-W., Wang M. (2024). Enhancing the bioavailability of quercetin via the construction of carboxymethylated curdlan/quercetin nanocomplex. Food Hydrocoll..

[6] Jiang F., Du C., Zhao N., Jiang W., Yu X., Du S.-K. (2022). Preparation and characterization of quinoa starch nanoparticles as quercetin carriers. Food Chem..

[7] McClements D.J. (2021). Advances in edible nanoemulsions: Digestion, bioavailability, and potential toxicity. Prog. Lipid Res..

[8] Sathishkumar P., Li Z., Govindan R., Jayakumar R., Wang C., Long Gu F. (2021). Zinc oxide-quercetin nanocomposite as a smart nano-drug delivery system: Molecular-level interaction studies. Appl. Surf. Sci..

[9] Qiu J., Chen X., Netrusov A.I., Zhou Q., Guo D., Liu X., He H., Xin X., Wang Y., Chen L. (2017). Screening and Identifying Antioxidative Components in Ginkgo biloba Pollen by DPPH-HPLC-PAD Coupled with HPLC-ESI-MS2. PLoS ONE.

[10] Shan H., Kong H. (2021). The genome of Ginkgo biloba refined. Nat. Plants.

[11] Zhang Z., Chen S., Mei H., Xuan J., Guo X., Couch L., Dobrovolsky V.N., Guo L., Mei N. (2015). Ginkgo biloba leaf extract induces DNA damage by inhibiting topoisomerase II activity in human hepatic cells. Sci. Rep..

[12] Wang T., Wu C., Fan G., Li T., Gong H., Cao F. (2018). Ginkgo biloba extracts-loaded starch nano-spheres: Preparation, characterization, and in vitro release kinetics. Int. J. Biol. Macromol..

[13] Wang T., Wu C., Li T., Fan G., Gong H., Liu P., Yang Y., Sun L. (2020). Comparison of two nanocarriers for quercetin in morphology, loading behavior, release kinetics and cell inhibitory activity. Mater. Express.

[14] Ghosh N., Sandur R., Ghosh D., Roy S., Janadri S. (2017). Acute, 28days sub acute and genotoxic profiling of Quercetin-Magnesium complex in Swiss albino mice. Biomed. Pharmacother..

[15] Tsuboi T., Hattori K., Ishimoto T., Imai K., Doke T., Hagita J., Ariyoshi J., Furuhashi K., Kato N., Ito Y. (2025). In vivo efficacy and safety of systemically administered serinol nucleic acid-modified antisense oligonucleotides in mouse kidney. Mol. Ther. Nucleic Acids.

[16] Lu Y., Wang R.H., Guo B.B., Jia Y.P. (2016). Quercetin inhibits angiotensin II induced apoptosis via mitochondrial pathway in human umbilical vein endothelial cells. Eur. Rev. Med. Pharmacol. Sci..

[17] Guo H., Ding H., Tang X., Liang M., Li S., Zhang J., Cao J. (2021). Quercetin induces pro-apoptotic autophagy via SIRT1/AMPK signaling pathway in human lung cancer cell lines A549 and H1299 in vitro. Thorac. Cancer.

[18] Gentile E.A., Castronuovo C.C., Cuestas M.L., Gómez N., Davio C., Oubiña J.R., Mathet V.L. (2019). F127 poloxamer effect on cytotoxicity induction of tumour cell cultures treated with doxorubicin. J. Pharm. Pharmacol..

[19] Yan Z., Wang Q., Liu X., Peng J., Li Q., Wu M., Lin J. (2018). Cationic nanomicelles derived from Pluronic F127 as delivery vehicles of Chinese herbal medicine active components of ursolic acid for colorectal cancer treatment. RSC Adv..

[20] Vendel E., Rottschäfer V., de Lange E.C.M. (2019). The need for mathematical modelling of spatial drug distribution within the brain. Fluids Barriers CNS.

[21] Kong T., Zhang S.H., Zhang C., Zhang J.L., Yang F., Wang G.Y., Yang Z.J., Bai D.Y., Shi Y.Y., Liu T.Q. (2020). Correction to: The Effects of 50 nm Unmodified Nano-ZnO on Lipid Metabolism and Semen Quality in Male Mice. Biol. Trace Elem. Res..

[22] Yang H., Wang M., Huang Y., Qiao Q., Zhao C., Zhao M. (2019). In vitro and in vivo evaluation of a novel mitomycin nanomicelle delivery system. RSC Adv..

[23] Akhter K.F., Mumin M.A., Lui E.M.K., Charpentier P.A. (2019). Immunoengineering with Ginseng Polysaccharide Nanobiomaterials through Oral Administration in Mice. ACS Biomater. Sci. Eng..

[24] Descotes J., Allais L., Ancian P., Pedersen H.D., Friry-Santini C., Iglesias A., Rubic-Schneider T., Skaggs H., Vestbjerg P. (2018). Nonclinical evaluation of immunological safety in Göttingen Minipigs: The CONFIRM initiative. Regul. Toxicol. Pharmacol..

[25] Wang T., Feng X., Li L., Luo J., Liu X., Zheng J., Fan X., Liu Y., Xu X., Zhou G. (2022). Effects of quercetin on tenderness, apoptotic and autophagy signalling in chickens during post-mortem ageing. Food Chem..

[26] Gu C., Stashko M.A., Puhl-Rubio A.C., Chakraborty M., Chakraborty A., Frye S.V., Pearce K.H., Wang X., Shears S.B., Wang H. (2019). Inhibition of Inositol Polyphosphate Kinases by Quercetin and Related Flavonoids: A Structure-Activity Analysis. J. Med. Chem..

[27] Pandey A.K., Shukla S.C., Bhattacharya P., Patnaik R. (2016). A possible therapeutic potential of quercetin through inhibition of μ-calpain in hypoxia induced neuronal injury: A molecular dynamics simulation study. Neural Regen. Res..

[28] Lu J., Papp L.V., Fang J., Rodriguez-Nieto S., Zhivotovsky B., Holmgren A. (2006). Inhibition of Mammalian thioredoxin reductase by some flavonoids: Implications for myricetin and quercetin anticancer activity. Cancer Res..

[29] Vijayababu M.R., Kanagaraj P., Arunkumar A., Ilangovan R., Aruldhas M.M., Arunakaran J. (2005). Quercetin-induced growth inhibition and cell death in prostatic carcinoma cells (PC-3) are associated with increase in p21 and hypophosphorylated retinoblastoma proteins expression. J. Cancer Res. Clin. Oncol..

[30] Latha R., Rajanathan T.M.C., Khusro A., Chidambaranathan N., Agastian P., Nagarajan S. (2019). Anticancer activity of Mahonia leschenaultii methanolic root extract and berberine on Dalton’s ascitic lymphoma in mice. Asian Pac. J. Trop. Med..

[31] Sun W., Zhao X., Fan J., Du J., Peng X. (2019). Boron Dipyrromethene Nano-Photosensitizers for Anticancer Phototherapies. Small.

[32] Zheng Y., Zhang J., Zhang R., Luo Z., Wang C., Shi S. (2019). Gold nano particles synthesized from Magnolia officinalis and anticancer activity in A549 lung cancer cells. Artif. Cells Nanomed. Biotechnol..

[33] Cunningham P., Patton E., VanderVeen B.N., Unger C., Aladhami A., Enos R.T., Madero S., Chatzistamou I., Fan D., Murphy E.A. (2022). Sub-chronic oral toxicity screening of quercetin in mice. BMC Complement. Med. Ther..

[34] Zhao Q., Yang Z.-S., Cao S.-J., Chang Y.-F., Cao Y.-Q., Li J.-B., Yao Z.-X., Wen Y.-P., Huang X.-B., Wu R. (2019). Acute oral toxicity test and assessment of combined toxicity of cadmium and aflatoxin B1 in kunming mice. Food Chem. Toxicol..

[35] Dolati P., Zamiri M.J., Akhlaghi A., Jahromi Z. (2021). P–060 Dose- dependent mitigation of lead acetate toxicity in males on embryo development in female mice. Hum. Reprod..

[36] Wang W., Sun C., Mao L., Ma P., Liu F., Yang J., Gao Y. (2016). The biological activities, chemical stability, metabolism and delivery systems of quercetin: A review. Trends Food Sci. Technol..

[37] Shen B., Zhu Y., Wang F., Deng X., Yue P., Yuan H., Shen C. (2024). Fabrication and in vitro/vivo evaluation of quercetin nanocrystals stabilized by glycyrrhizic acid for liver targeted drug delivery. Int. J. Pharm. X.

[38] Hwang S., Koo I., Patterson A.D., Lambert J.D. (2023). Comparative urine metabolomics of mice treated with non-toxic and toxic oral doses of (−)-epigallocatechin-3-gallate. Food Funct..

[39] Akal Z.Ü., Alpsoy L., Baykal A. (2016). Biomedical applications of SPION@APTES@PEG-folic acid@carboxylated quercetin nanodrug on various cancer cells. Appl. Surf. Sci..

[40] Ekambaram P., Parasuraman P., Jayachandran T. (2016). Differential regulation of pro- and antiapoptotic proteins in fish adipocytes during hypoxic conditions. Fish Physiol. Biochem..

[41] Zhang Y., Xu X., He P. (2011). Tubeimoside-1 inhibits proliferation and induces apoptosis by increasing the Bax to Bcl-2 ratio and decreasing COX-2 expression in lung cancer A549 cells. Mol. Med. Rep..

[42] Flores-Romero H., Hohorst L., John M., Albert M.C., King L.E., Beckmann L., Szabo T., Hertlein V., Luo X., Villunger A. (2022). BCL-2-family protein tBID can act as a BAX-like effector of apoptosis. EMBO J..

[43] Almannai M., Salah A., El-Hattab A.W. (2022). Mitochondrial Membranes and Mitochondrial Genome: Interactions and Clinical Syndromes. Membranes.

[44] Shi S.-W., Li Y.-H., Zhang Q.-L., Yang S.-P., Liu J.-G. (2019). Targeted and NIR light-controlled delivery of nitric oxide combined with a platinum(iv) prodrug for enhanced anticancer therapy. J. Mater. Chem. B.

[45] Ozturk N., Ozturk D., Pala-Kara Z., Kaptan E., Sancar-Bas S., Ozsoy N., Cinar S., Deniz G., Li X.M., Giacchetti S. (2018). The immune system as a chronotoxicity target of the anticancer mTOR inhibitor everolimus. Chronobiol. Int..

[46] Miao T., Wang J., Zeng Y., Liu G., Chen X. (2018). Polysaccharide-Based Controlled Release Systems for Therapeutics Delivery and Tissue Engineering: From Bench to Bedside. Adv. Sci..

[47] Zhang L., Kuang G., Gong X., Huang R., Zhao Z., Li Y., Wan J., Wang B. (2024). Piperine attenuates hepatic ischemia/reperfusion injury via suppressing the TLR4 signaling cascade in mice. Transpl. Immunol..

[48] He Z., Zhang L., Li Z., Gao X., Zhang Y., Gao F. (2025). Quantitative Detection of Orthotopic Liver Cancer in Mice Using Indocyanine Green and Dynamic Diffuse Fluorescence Tomography Imaging. J. Biophotonics.

