# Correction: Sun et al. Quercetin-Loaded Ginkgo Starch Nanoparticles: A Promising Strategy to Improve Bioactive Delivery and Cellular Homeostasis in Functional Foods. *Foods* 2025, *14*, 1890

**DOI:** 10.3390/foods15010004

**Published:** 2025-12-19

**Authors:** Yanyu Sun, Kaiping Cong, Tao Wang, Xiaojing Li, Tingting Li, Gongjian Fan, Dandan Zhou, Caie Wu

**Affiliations:** 1 National Key Laboratory for the Development and Utilization of Forest Food Resources, College of Light Industry and Food Engineering, Nanjing Forestry University, Nanjing 210037, China; 2 Affiliated Cixi Hospital, Wenzhou Medical University, Cixi 315300, China; 3 Department of Chemical Engineering, Xuzhou College of Industrial Technology, Xuzhou 221140, China

## Error in Affiliation

In the published publication [1], there was an error regarding the affiliation 3 for Tao Wang. The updated affiliation 3 should be: Department of Chemical Engineering, Xuzhou College of Industrial Technology, Xuzhou 221140, China. The authors state that the scientific conclusions are unaffected. This correction was approved by the Academic Editor. The original publication has also been updated.
