# Supplementary material for: Quercetin-Loaded Ginkgo Starch Nanoparticles: A Promising Strategy to Improve Bioactive Delivery and Cellular Homeostasis in Functional Foods
Source: Foods. 2025 May 26;14(11):1890. doi: 10.3390/foods14111890 (PMC12155164; doi:10.3390/foods14111890)
Supplement: Supplementary file 1 [file foods-14-01890-s001.zip › foods-3622558-Supplementary.pdf]

Supplementary data

Table S1. IC50 of Qc, SNPs/Qc and F127/Qc on five kinds of cancer cells.

Sample	IC50 Value (μg/mL)				
	**3LL**	**HEPG-2**	**BGC-823**	**MCF-7**	**HCT116**
Qc	179.10	1730.60	2690.67	1785.10	17934.92
SNPs/Qc	64.50	2221.48	923.40	685.88	1714.45
F127/Qc	43.50	238.51	352.08	763.57	1956.16

Table S2. Effect of SNPs/Qc and F127/Qc on 3LL cell apoptosis.

Group	Dose (μg/ml)	UL (%)	UR (%)	LL (%)	LR (%)	Apoptosis (%)
CK	0	0.98 ± 1.12^{b}	1.57 ± 0.18^{b}	94.91 ± 2.76^{a}	2.54 ± 0.02d	4.11 ± 0.45d
	6.25	1.10 ± 0.31^{b}	8.38 ± 0.78^{b}	74.09 ± 4.01^{c}	12.44 ± 1.56^{c}	20.82 ± 2.34^{c}
SNPs/Qc	12.5	0.80 ± 0.17^{b}	6.16 ± 1.36^{c}	67.42 ± 2.16^{d}	22.62 ± 1.01^{b}	28.78 ± 2.37^{b}
	25	1.11 ± 0.02^{b}	4.01 ± 2.08^{cd}	52.84 ± 2.47^{e}	33.04 ± 2.59^{a}	37.05 ± 1.91^{a}
	6.25	0.59 ± 0.22^{c}	10.47 ± 1.03^{a}	79.93 ± 3.78^{b}	11.00 ± 1.17^{c}	21.47 ± 2.93^{c}
F127/Qc	12.5	0.84 ± 0.31^{b}	8.90 ± 1.26^{b}	67.84 ± 2.90^{d}	22.42 ± 2.23^{b}	31.32 ± 2.81^{b}
	25	1.45 ± 0.21^{a}	6.13 ± 2.10^{c}	51.07 ± 3.20^{e}	34.35 ± 2.03^{a}	40.48 ± 4.04^{a}

Means with the different lowercase letters in the same column are significantly different according to ANOVA test ($p < 0.05$).

Table S3. Effect of SNPs/Qc and F127/Qc on 3LL cell cycle.

Parameters	Dose (μg/ml)	G1 Phase (%)	S Phase (%)	G2 Phase (%)
CK	0	46.56 ± 0.54[a]	40.93 ± 1.01[a]	12.51 ± 0.28[d]
	6.25	39.96 ± 0.88[c]	40.14 ± 0.68[a]	19.90 ± 0.39[b]
SNPs/Qc	12.5	39.37 ± 1.64[c]	39.80 ± 0.37[a]	20.83 ± 1.02[b]
	25	35.90 ± 0.73[d]	40.26 ± 1.11[a]	23.84 ± 0.54[a]
	6.25	43.03 ± 1.36[b]	39.30 ± 1.09[a]	17.67 ± 0.73[c]
F127/Qc	12.5	40.57 ± 1.69[c]	38.84 ± 1.47[ab]	20.60 ± 0.21[b]
	25	37.86 ± 1.08[c]	38.06 ± 0.23[b]	24.08 ± 0.82[a]

Means with the different lowercase letters in the same column are significantly different according to ANOVA test ($p < 0.05$).

Table S4. Effect of SNPs/Qc and F127/Qc on mitochondrial membrane potential of 3LL cell.

Parameters	Dose (μg/ml)	Positive cell (%)
CK	0	3.72 ± 0.21[g]
	6.25	18.72 ± 1.11[f]
SNPs/Qc	12.5	32.86 ± 2.10[d]
	25	42.40 ± 1.92[b]
	6.25	20.95 ± 1.38[e]
F127/Qc	12.5	36.71 ± 1.97[c]
	25	47.68 ± 3.01[a]

Means with the different lowercase letters in the same column are significantly different according to ANOVA test ($p < 0.05$).
